# Feasible Structure Manipulation of Vanadium Selenide into VSe_2_ on Au(111)

**DOI:** 10.3390/nano12152518

**Published:** 2022-07-22

**Authors:** Chaoqin Huang, Lei Xie, Huan Zhang, Hongbing Wang, Jinping Hu, Zhaofeng Liang, Zheng Jiang, Fei Song

**Affiliations:** 1 Shanghai Institute of Applied Physics, Chinese Academy of Sciences, Shanghai 201000, China; huangchaoqin@sinap.ac.cn (C.H.); zhanghuan@sinap.ac.cn (H.Z.); wanghongbing@sinap.ac.cn (H.W.); hujinping@sinap.ac.cn (J.H.); jiangzheng@sinap.ac.cn (Z.J.); 2 University of Chinese Academy of Sciences, Beijing 101000, China; 3 Shanghai Synchrotron Radiation Facility, Shanghai Advanced Research Institute, Chinese Academy of Sciences, Shanghai 201204, China; sleung1924@gmail.com

**Keywords:** transition metal dichalcogenides, phase transition, scanning tunneling microscopy, controlled structural manipulation, density functional theory calculations

## Abstract

Vanadium diselenide (VSe_2_), a member of the transition metal dichalcogenides (TMDs), is proposed with intriguing properties. However, a comprehensive investigation of VSe_2_ (especially regarding on the growth mechanism) is still lacking. Herein, with the molecular beam epitaxy (MBE) measures frequently utilized in surface science, we have successfully synthesized the single-layer VSe_2_ on Au(111) and revealed its structural transformation using a combination of scanning tunneling microscopy (STM) and density functional theory (DFT). Initially, formation of the honeycomb structure is observed with the moiré periodicity, which is assigned to VSe_2_. Followed by stepwise annealing, defective structures with streaked patterns start to emerge due to the depletion of Se, which can be reversed to the pristine VSe_2_ by resupplying Se. With more V than Se deposited, a new compound that has no bulk analogue is discovered on Au(111), which could be transformed back to VSe_2_ after providing excessive Se. As the realization of manipulating V selenide phases is subtly determined by the relative ratio of V to Se and post-annealing treatments, this report provides useful insights toward fundamental understanding of the growth mechanism of TMDs and might promote the wide application of VSe_2_ in related fields such as catalysis and nanoelectronics.

## 1. Introduction

Transition metal dichalcogenides (TMDs) with the typical sandwich structure, are some of the most promising two-dimensional (2D) materials possessing unique electronic, magnetic and mechanical properties, and have been subjected to intense investigations in the past decades. In practice, TMDs have a general formula: MX_2_ [1,2], where M represents transition metal elements (M = Ti, V, Ta, Mo, Re, W and so on) and X represents the chalcogen atom (X = S, Se, Te). The MX_2_ crystal structure is considered to own strong intralayer covalent coupling (M-X) and weak van der Waals interactions between stacked layers due to large interlayer spacing (0.6–0.7 nm) [1,3]. Thanks to these unique structural configurations, TMDs have been discovered with versatile electronic properties [4,5] from semiconductors (e.g., MoS_2_, WSe_2_), metallic behavior (e.g., 1T MoTe_2_, VSe_2_), to superconductors (e.g., NbS_2_, NbSe_2_) [1,6] and magnetic materials [7] in literature reports. Consequently, TMDs becomes promising candidates in numerous applications, such as nanoelectronics [8,9], highly active catalysts [10,11,12,13,14], novel sensors [15], energy storage [16,17] and so forth.

Specifically, vanadium diselenide (VSe_2_), as one of the important members in the TMDs family, has also been credited with unique properties such as high conductivity [18,19], 2D ferromagnetism [20,21,22,23], charge density wave (CDW) [24,25,26], and excellent electrocatalytic activities [27,28]. In practice, ultrathin sheets of TMDs have been fabricated via the mechanical exfoliation, colloidal synthesis [29], chemical vapor deposition (CVD) [30,31,32,33,34] or liquid-phase exfoliation [35,36]. Following these strategies, structural defects and contamination are inevitably introduced in synthesized TMDs, which impede the fine characterization of the corresponding electronic structures and physical properties. Therefore, fabrication of VSe_2_ utilizing surface science techniques was proposed, and VSe_2_ layers have been successfully grown on highly oriented pyrolytic graphite (HOPG) or MoS_2_ surfaces [22,37,38,39]. However, the construction of single-layer (SL) VSe_2_ on metal substrates (for instance, gold) has been scarcely mentioned in literature and related structure investigation is thus rare. On the other hand, TMDs on metals just represents an important concern in 2D electronic devices, as atomically thick TMDs have to be connected with metals that serve as electrodes or connectors for device applications.

In this context, we have chosen gold as substrate which is known to form vdW interfaces with TMDs due to its chemical stability [40], and successfully synthesized the single-layer (SL) VSe_2_ on Au(111) via molecular beam epitaxy (MBE) in this report and elaborately investigated the structural transformation of V selenide by utilizing a combination of STM and density functional theory (DFT) calculations. Controllable fabrication of VSe_2_ and its defect structures is achieved by subtle regulation of deposition ratios and thermal treatment temperatures. Such investigation reveals the dynamic nature of monolayer VSe_2_ on Au(111) by STM, provides valuable insights into the synthesis and characterization of TMDs and their defect structures.

## 2. Materials and Methods

STM measurements were performed in an ultrahigh vacuum system (SPECS-NAP150, Berlin, Germany) with a base pressure better than 2 × 10^−10^ mbar [41]. All STM images were acquired at room temperature in the constant-current mode and processed afterwards by the WSXM software (4.0 Beta) [42]. A clean and structurally well-defined Au(111) surface was obtained through cycles of sputtering with Ar^+^ ions and subsequent annealing at 450 °C, as judged by the presence of the herringbone reconstruction [43]. Single layer VSe_2_ was grown on Au(111) via simultaneously evaporating V and excessive Se from a customized electron-beam evaporator (ACME, Shanghai, China) [44] and an evaporation source in the preparation chamber with the base pressure of 1 × 10^−9^ mbar, while the substrate was held at 300 °C during deposition. The scanning process was carried out on many different spots over the sample, where majority of surface species (approximately 90%) can be assigned to the corresponding target structures.

Density functional theory (DFT) calculations were performed by using the Vienna Ab initio Simulation Package (VASP) [45]. The projector augmented wave (PAW) [46] was employed and the Perdew–Burke–Ernzerhof (PBE) was utilized in the framework of generalized gradient approximation (GGA) [47] and the dispersion corrected DFT-D3 method of Grimme [48] was used for the calculations when including the weak interactions. The electronic wave functions were expanded in plane waves with the energy cutoff of 500 eV. During structure relaxation, the bottom two Au layers from substrate were fixed while all the other atoms were free to relax until the atomic force was less than 0.05 eV/Å [49].

## 3. Results and Discussion

**Figure 1:**
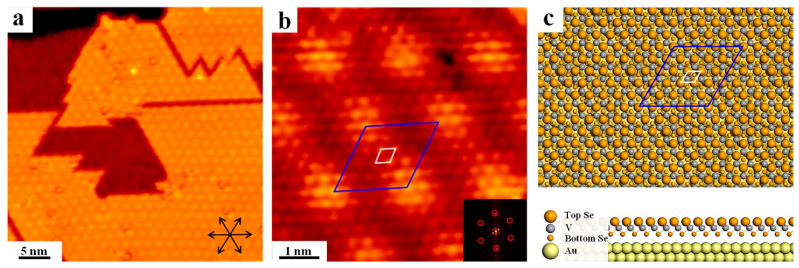
(**a**) The overview STM of the V selenide grown on Au(111). Black arrows: high-symmetry directions of the Au(111) substrate. (**b**) High-resolution STM with the moiré pattern resolved. Unit cells of the V selenide as well as the moiré pattern are indicated by colored rectangles, while FFT is shown inset indicating the periodicity of moiré structure. (**c**) DFT predicated configuration of VSe_2_ on Au(111) with top and side views. Au: yellow; Se: green; V: red. Scanning parameters: (**a**) U_bias_ = −1.0 V, I_tunneling (t)_ = 0.6 nA; (**b**) U_bias_ = −1.2 V, I_t_ = 1.2 nA.

Initially, simultaneous deposition of V and excessive Se onto Au(111) held at 300 °C has been performed (with the V/Se ratio at about 1:10), and the well-ordered superstructure in triangular islands is discovered as presented in Figure 1a, which is in fact analogous to the typical appearance of layered TMDs on metal surfaces [50]. Nevertheless, gaps can be resolved between islands that have almost merged together at this coverage. It is revealed from the atomically resolved STM in Figure 1b that the quasi-triangle domain is constructed in a close packed pattern with the hexagonal structure, and the distance between neighboring dots is found to be around 0.35 nm, as the atomic lattice is highlighted by the white rhombus in Figure 1b. Besides, the moiré structure is also observed with a much larger superlattice, and the periodicity is measured to be about 1.77 nm with the primary unit cell delineated by the blue rhombus in Figure 1b. Based on these observations, it might be inferred that the island is only one layer in thickness with the consideration of both the apparent height with respect to the Au surface 0.184 nm (Figure S1c), and the clear appearance of the moiré superstructure, which is usually ambiguous in bilayer [51,52,53]. Consequently, the close packed structure shall be assigned to the formation of single layer (SL) VSe_2_ after comparison with previous studies [22,37,54,55], where Se atoms on the top layer are imaged as bright dots in STM. Notably, the lattice mismatch between the as-grown V selenide and the underlying Au(111) substrate induces the emergence of the apparent moiré corrugation with a (5 × 5) superlattice, which is commensurate with a (6 × 6) reconstruction of the underlying Au(111). Notably, the atomic lattice constant herein is apparently larger than reported values in literature [56], which shall be assigned to the substrate influence during epitaxy growth to achieve a commensurate interfacial structure. Besides, fast Fourier transform (FFT) of STM image is also displayed as an inset showing the relative ratio of lattice between VSe_2_ and the moiré pattern.

In order to further investigate the atomic structure of VSe_2_ on Au(111), DFT calculations have also been performed on the basis of STM data. The optimized configuration is illustrated in Figure 1c. In general, the monolayer VSe_2_ adsorbed on Au(111) shows a hexagonal lattice, whereas the V is sandwiched between two layer Se from top and bottom. Since STM topography at RT is not capable of distinguishing the difference between 1T or 2H phases of VSe_2_, just the 1T structure is chosen herein for discussion for simplicity. The distance between adjacent superficial Se atoms is calculated to be 0.34 nm, and the moiré unit cell is revealed to be 1.73 nm, which agrees reasonably with the experimental data. Furthermore, the moiré periodicity corresponding to a (5 × 5) supercell fits well onto 6 unit cells of Au(111). Therefore, it can be confirmed that the moiré corrugation is induced by the slight lattice difference between VSe_2_ and Au(111), while analogous observations have also been demonstrated for SL MoS_2_ on Au(111) with the moiré periodicity of 2.30 nm [57,58,59]. It is also worth pointing out that, varying deposition sequence of V and Se (the same sublimation ratio as before) have also been implemented. Interestingly, the same moiré structure has always been obtained on Au(111) regardless of deposition sequence, which are represented in Figure S2. These observations suggest that VSe_2_ with the moiré phase is indeed a fairly stable phase on Au(111).

**Figure 2:**
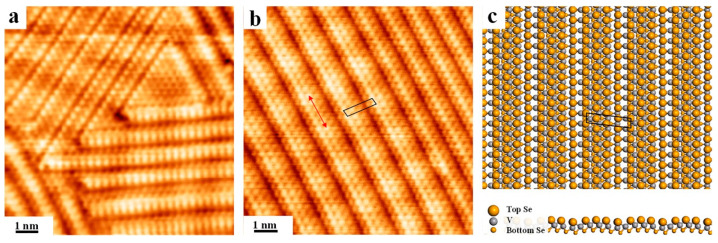
Formation of the streaked structure after annealing the pristine VSe_2_ at 450 °C. (**a**) STM image of linear chains with three orientations corresponding to the three-fold symmetry of Au(111). (**b**) Atomically resolved STM of the striped pattern with two different periodicities. (**c**) Configuration for the typical streaked structure from DFT calculations with top and side views. The unit cell is highlighted by the black parallelogram. Scanning parameters: (**a**) U_bias_ = 1.2 V, I_t_ = 5.6 nA; (**b**) U_bias_ = −1.2 V, I_t_ = 3.0 nA.

To further explore the thermal stability of VSe_2_ grown on Au(111), post-annealing has also been conducted for the VSe_2_-Au(111) substrate. After annealing to 350 °C, defects start to emerge in the moiré phase as seen in Figure S3b. Dislocation lines are apparent (indicated by black arrows) disrupting the long-range periodicity and are presumably caused by the initiation of the phase change. Further annealing to 400 °C leads to a transition of the moiré structure to yet a distorted phase (Figure S3c), whose signature in STM is the striped appearance. As discovered, the moiré pattern is substantially destroyed by these striped lines, while the moiré corrugation can still be identified at certain areas. Complete transition of the pristine hexagonal structure of VSe_2_ is seen after annealing at 450 °C, and is represented in Figure 2a. Interestingly, the long-range ordered structure with alternating bright strips is revealed, while streaks owing varying widths are aligned along the main symmetry directions of the underlying Au(111). A rectangular unit cell is identified for one of streaked phases (highlighted by black lines) with lattice parameters of 1.36 nm and 0.31 nm from the atomically resolved STM image in Figure 2b. As seen, the unit cell contains four rows of alternating bright and dim atoms with the apparent height difference by about 0.048 nm (Figure S1d), while the dim appearance might be related to the desorption of Se after intense annealing. Moreover, intriguingly, the moiré periodicity can still be recognized along streaks with the dimension preserved (1.77 nm), as marked by red arrows in Figure 2b, whilst the distance of moiré protrusions between neighboring streaks is shrunk from the pristine value due to the structural transition. In practice, it has also been demonstrated in the previous report that, annealing the as-grown VSe_2_ on HOPG can induce the conversion of the pristine hexagonal phase into chain-like structures due to the depletion of Se [60]. Thus, it is inferred herein that the close packed VSe_2_ is converted to a defective structure with stripped appearances after annealing to 450 °C as a result of the regular loss of Se in columns from the superficial layer.

To further understand the streaked pattern formed after annealing in Figure 2b (marked with black lines), the corresponding configuration in gas phase has also been constructed from DFT calculations. As seen in Figure 2c, with one missing column of Se atoms on average five columns, the optimized structural model reasonably reproduces the streaked pattern, and the unit cell parameters are calculated to be 1.39 nm by 0.32 nm, which are in agreement with the STM measurement. Because of the selective desorption of Se in column, atoms located at the missing row is thus lower than neighboring superficial Se atoms by about 0.07 nm, leading to the dim appearance in STM imaging. Nevertheless, the unit cell vector of 1.39 nm in the long direction is shorter than the original value (1.73 nm) after the depletion of Se due to shrinking, and the moiré periodicity along streaks is somehow retained.

Structural transition has been observed induced by the gradual depletion of Se atoms after sequential annealing, while redosing sufficient Se atoms into the defective structure might bring the pristine hexagonal VSe_2_ phase back, as demonstrated before in the VS_2_ system [61]. Under this consideration, Se was redeposited onto the stripped structure while the substrate is still kept at 300 °C. As expected, close packed structure with the homogeneous pattern is recovered after redeposition, as illustrated in Figure S4. Supported by the observation that the annealing induced phase changes can be reversed by annealing in the Se atmosphere, consequently, the previous hypothesis can be confirmed that streaked structures are caused by the depletion of Se atoms due to the gradual desorption after thermal annealing. More interestingly, the reversible structural transition is easily manipulated between the hexagonal phase and striped pattern by depletion and resupplying of Se via the approach of post annealing and redeposition.

**Figure 3:**
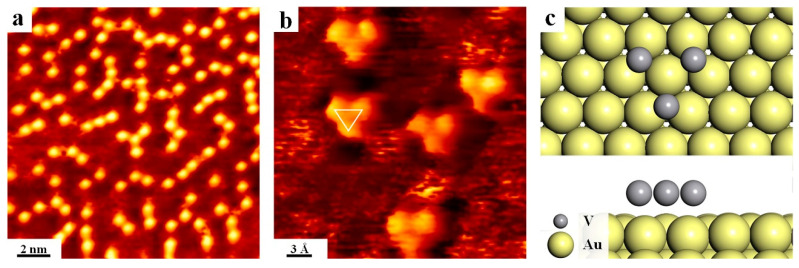
The observation of clusters formation on Au(111) after annealing at 480 °C. (**a**) The overview STM. (**b**) Zoom-in view showing the identical adsorption configuration. (**c**) Top and side views of the optimized model of the V cluster on Au(111). The cluster is marked with the white triangle. Scanning parameters: (**a**) U_bias_ = −0.9 V, I_t_ = 0.6 nA; (**b**) U_bias_ = −0.6 V, I_t_ = 4.2 nA.

Further annealing to 480 °C was implemented afterwards based on streaked structures, and it is revealed that surface is decorated with randomly distributed bright clusters as shown in Figure 3a, which is most probably contributed by the increased loss Se atoms. The zoom-in view of clusters is shown in Figure 3b, and one can easily find that the cluster is actually composed of three bright dots packed in an inverted triangle pattern (indicated by white lines) with lattice parameters of 0.33 nm. These clusters can only be assigned to the V related species as a result of continuous loss of Se and the relative high activity of V on Au [62], namely, three V atoms bound together by adsorption on Au(111). Meanwhile, DFT calculations have also been conducted to figure out the possible structure for these clusters. The most possible configuration is shown in Figure 3c from the energy point of view, whereas each V atom is located at the hollow site on Au(111), whereby a lattice unit cell is depicted with the lattice constant calculated to be 0.37 nm. Consequently, we can conclude that only V atoms remains on Au(111) after intensive annealing to 480 °C, and the pristine VSe_2_ almost decompose at this stage, which has been seldom mentioned in literature.

**Figure 4:**
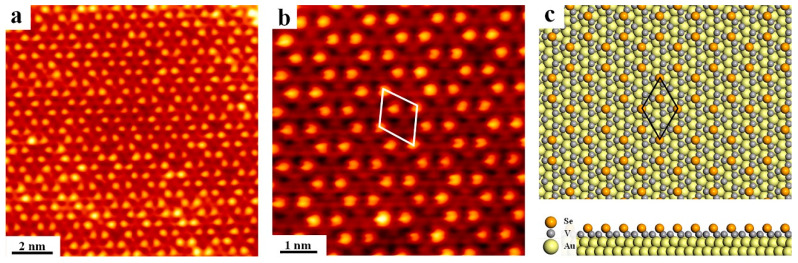
The hexagon-like pattern formed on Au(111) with a varied ratio of V to Se. (**a**) The overview and (**b**) zoom-in view of the hexagonal defect structures with the white rhombus highlighting the unit cell. (**c**) Top and side views of the relaxed model of the hexagonal structures. Scanning parameters: (**a**) U_bias_ = −1.5 V, I_t_ = 0.92 nA; (**b**) U_bias_ = −1.5 V, I_t_ = 1.05 nA.

Besides the manipulation of thermal annealing, varying the relative ratio of V to Se has also been explored to investigate the structure variation of V selenide. Relatively sufficient V with a V:Se ratio of 2:1 were deposited onto Au(111) kept at 300 °C, and appearance of the hexagonal-like pattern is found with long-range order, as shown in Figure 4a. Due to the relative sufficiency of V and deficiency of Se, the newly formed phase shall be related to a V-rich structure that has no bulk analogue. Revealed from the zoom-in STM image in Figure 4b, the hexagonal pattern is actually constructed with regularly alternating bright and dim dots. It is primarily inferred that V atoms are first close packed in array (triangular shape) on Au(111) as dim dots in STM while Se atoms are selectively adsorbed afterwards on top of V arrays as bright protrusions, resulting in the nearest Se-Se distance of 0.55 nm, and a lattice unit cell (white lines) is depicted with the lattice constant measured to be 0.91 nm. As Se is missing in every third V arrays, it can be concluded that the compound is fabricated with the V_9_Se_2_ stoichiometry in the end. Moreover, the DFT optimized configuration has also been obtained and is shown in Figure 4c, and the lattice unit cell is marked with the lattice parameter calculated to be 0.94 nm, which is in principle consistent with the STM result. In addition, the Se atom is predicted to be adsorbed on the hollow site of the V array, giving a reasonable arrangement of V_9_Se_2_ on top of Au(111).

To further verify the rationality of the proposed V selenide structure, redepositing Se was carried out with the substrate kept at 300 °C. Intriguingly, the hexagonal VSe_2_ structure was brought back as shown in Figure S5, proving that the previous V selenide structure is indeed related with the deficiency of Se. However, this hexagon-defect phase cannot be obtained by solely annealing the pristine VSe_2_ structure under UHV, implying that such structural transformation is irreversible, in contrast to the previously observed phenomenon that streaked structures can be achieved by thermal annealing VSe_2_ at elevated temperatures and vice versa. This difference may also suggest that this V selenide compound is metastable from the thermodynamic point of view.

In summary, various V selenide complexes have been obtained on Au(111) by varying deposition parameters and post annealing treatments. While SL VSe_2_ can only be achieved on the gold surface in the condition of excessive Se relative to V, it is also feasible to convert VSe_2_ into defective architectures with streaked phase by post annealing and reverse them into VSe_2_ by readding more Se. On the other hand, insufficient supply of Se during MBE growth leads to the formation of another hexagon-like structure, which can be further tuned into SL VSe_2_ by adding more Se. Therefore, it is inferred that Se plays an important role in the construction of VSe_2_ related structures on Au(111) which is an ideal platform to fabricate vdW coupled TMDs atop, and this discovery may open a new channel to build artificial defects in TMDs which can function as active sites for physical chemistry reactions on the surface.

## 4. Conclusions

Fabrication of single-layer VSe_2_ on Au(111) has been realized with the controllable structural manipulation of vanadium selenide compounds, and a comprehensive investigation of structural transition mechanism has also been performed by combing STM and DFT calculations. Simultaneous deposition of vanadium with excessive selenium on Au(111) held at 300 °C results in the formation of the homogeneous SL VSe_2_. With sequential annealing at elevated temperatures, structural transition to streaked patterns with alternating bright protrusions is observed due to the selective loss of Se atoms. Intriguingly, the defective phase can be converted back to the pristine VSe_2_ by resupplying Se, manifesting the structural reversibility between vanadium selenide compounds. Altering the sublimation ratio of V/Se to 2:1, a hexagon-defect structure is formed on Au(111), which does not have a bulk analogue and can also be transformed to the hexagonal VSe_2_ by adding sufficient Se. Since the feasible structural manipulation of V-Se compounds has been witnessed, this work would promote a step forward towards the controllable fabrication of low dimensional TMDs materials with tuned defects and electronic structures on demand which might pose promising applications in physical and catalytic fields.

## Data Availability

The data presented in this study are available on request from the corresponding author.

## Supplementary Materials

The following supporting information can be downloaded at: https://www.mdpi.com/article/10.3390/nano12152518/s1, Figure S1: (a) The overview STM image of the VSe_2_ on Au(111). (b) STM image of linear chains. (c) The line profile along the black line in panel a. (d) The line profile collected along the black line in panel b; Figure S2: High resolution STM images showing the synthesis procedure of VSe_2_ on Au(111). Adding V atoms on Se-decorated Au(111) followed by annealing at 300 °C, or depositing Se on V-covered Au(111) at 300 °C both can lead to the formation of VSe_2_; Figure S3: High resolution STM images manifesting varying structures formed after annealing at elevated temperatures. (a) The as-synthesized VSe_2_ on Au(111), (b) annealing at 350 °C resulting in the emergence of defective lines as indicated by black arrows, (c) annealing at 400 °C bringing more the linear chains; (d) After annealing at 450 °C, the pristine VSe_2_ is in principle completed transformed into a new phase with the stripped pattern; Figure S4: STM recording of the reverse structural transformation from chain-like pattern to the homogeneous VSe_2_ after redepositing Se while the substrate is kept at 300 °C; Figure S5: Transition from the hexagon-like structure with ordered defects to the pristine homogeneous VSe_2_ via deposition of excessive Se atoms with the substrate at 300 °C.
