# Supplementary material for: Feasible Structure Manipulation of Vanadium Selenide into VSe2 on Au(111)
Source: Nanomaterials (Basel). 2022 Jul 22;12(15):2518. doi: 10.3390/nano12152518 (PMC9332180; doi:10.3390/nano12152518)
Supplement: Supplementary file 1 [file nanomaterials-12-02518-s001.zip › nanomaterials-1791978-supplementary.pdf]

nanomaterials

MDPI

Supplementary Materials

Feasible Structure Manipulation of Vanadium Selenide into VSe_2 on Au(111)

Chaoqin Huang [1,2], Lei Xie [3,*], Huan Zhang [1,2], Hongbing Wang [1,2], Jinping Hu [1,2], Zhaofeng Liang [3], Zheng Jiang [1,2,3] and Fei Song [1,2,3,*]

1 Shanghai Institute of Applied Physics, Chinese Academy of Sciences, Shanghai 201000, China; huangchaoqin@sinap.ac.cn (C.H.); zhanghuan@sinap.ac.cn (H.Z.); wanghongbing@sinap.ac.cn (H.W.); hujinping@sinap.ac.cn (J.H.); jiangzheng@sinap.ac.cn (Z.J.)
2 University of Chinese Academy of Sciences, Beijing 101000, China
3 Shanghai Synchrotron Radiation Facility, Shanghai Advanced Research Institute, Chinese Academy of Sciences, Shanghai 201204, China; sleung1924@gmail.com
* Correspondence: xiel@sari.ac.cn (L.X.); songfei@sinap.ac.cn (F.S.)

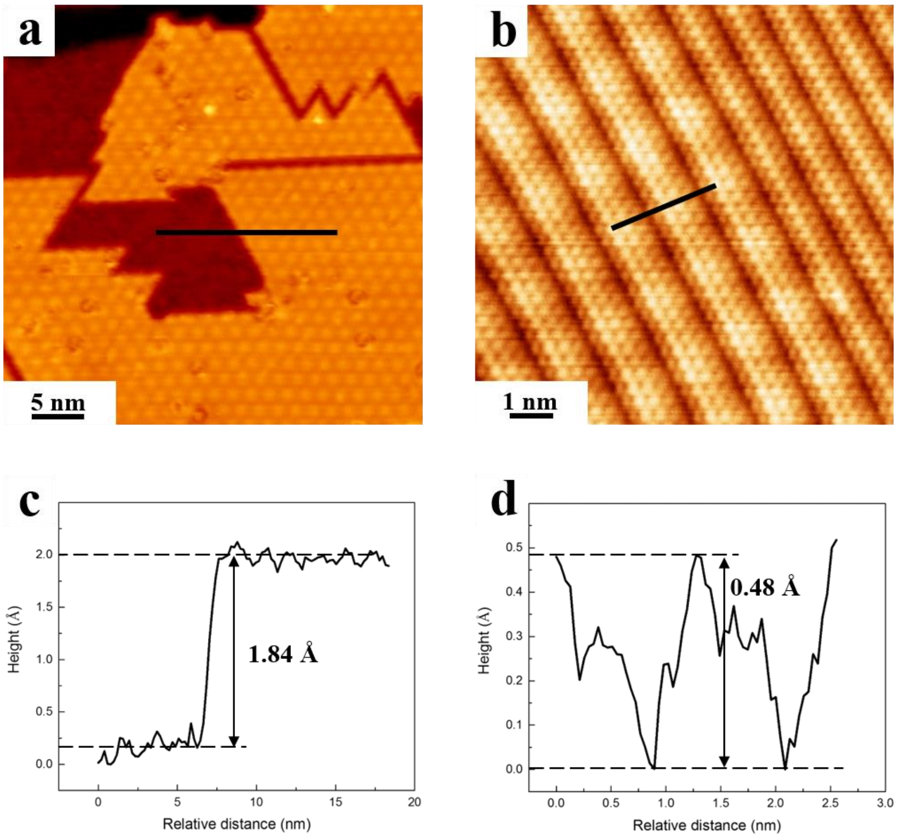

Figure S1. (**a**) The overview STM image of the VSe_2 on Au(111). (**b**) STM image of linear chains. (**c**) The line profile along the black line in panel a. (**d**) The line profile collected along the black line in panel b.

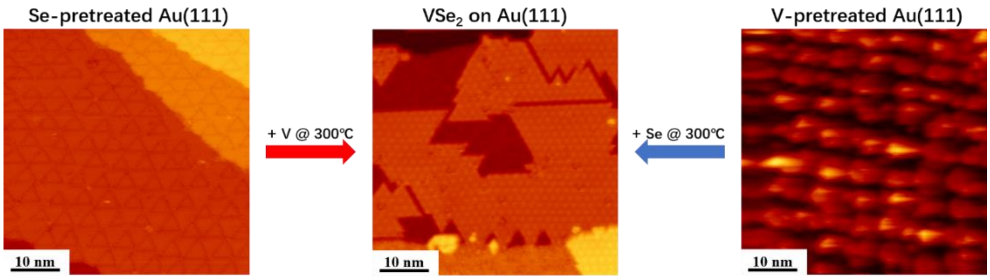

Figure S2. High resolution STM images showing the synthesis procedure of VSe_2 on Au(111). Adding V atoms on Se-decorated Au(111) followed by annealing at 300 °C, or depositing Se on V-covered Au(111) at 300°C both can lead to the formation of VSe_2.

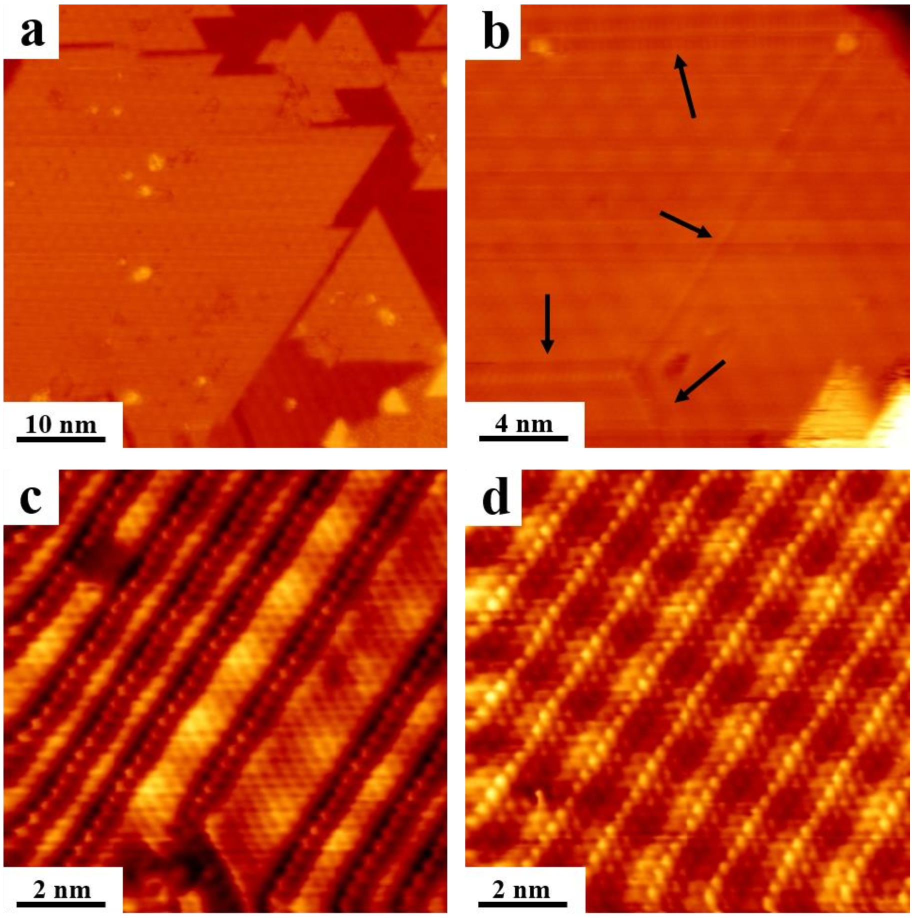

Figure S3. High resolution STM images manifesting varying structures formed after annealing at elevated temperatures. (**a**) The as-synthesized VSe_2 on Au(111), (**b**) annealing at 350 °C resulting in the emergence of defective lines as indicated by black arrows, (**c**) annealing at 400°C bringing more the linear chains; (**d**) After annealing at 450 °C, the pristine VSe_2 is in principle completed transformed into a new phase with the stripped pattern.

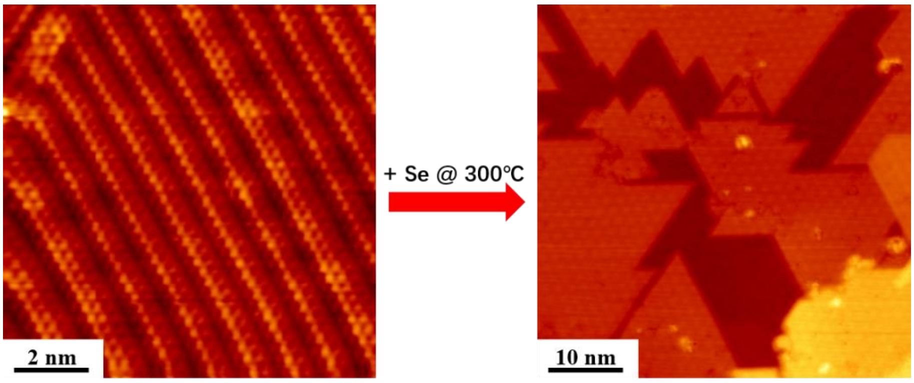

Figure S4. STM recording of the reverse structural transformation from chain-like pattern to the homogeneous VSe_2 after redepositing Se while the substrate is kept at 300 °C.

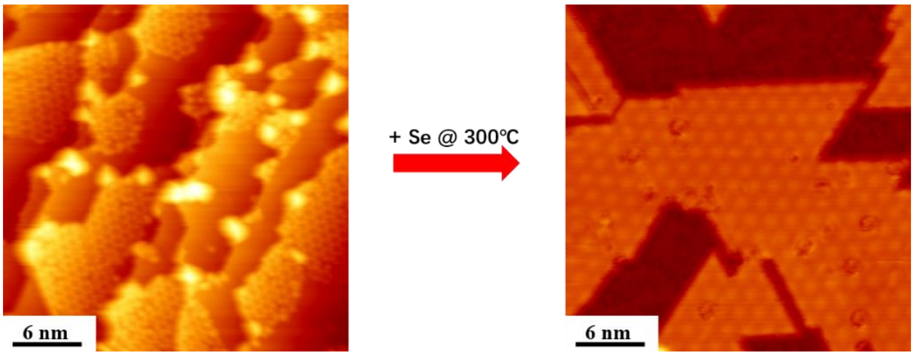

Figure S5. Transition from the hexagon-like structure with ordered defects to the pristine homogeneous VSe_2 via deposition of excessive Se atoms with the substrate at 300°C.
